# Reversible cerebral vasoconstriction syndrome post-cardiac transplantation: a therapeutic dilemma: case report

**DOI:** 10.1186/s12883-024-03780-3

**Published:** 2024-08-09

**Authors:** Natalie L. Montarello, Iain Irvine, Victoria Warner, James Hare, David Kaye, Geoffrey C. Cloud

**Affiliations:** 1 Department of Cardiology, The Alfred, Melbourne, VIC Australia; 2 Department of Radiology, The Alfred, Melbourne, VIC Australia; 3 Department of Pharmacy, The Alfred, Melbourne, VIC Australia; 4 Department of Stroke, The Alfred, Melbourne, VIC Australia

**Keywords:** Case report, Cerebral, Vasoconstriction, Transplantation, Tacrolimus

## Abstract

**Background:**

Reversible cerebral vasoconstriction syndrome (RCVS) is characterized by diffuse, multifocal segmental narrowing of cerebral arteries and can result in ischaemic stroke. Causal factors, identified in 60% of cases, include immunosuppressant pharmacotherapy. The few reports following heart transplantation are almost all in Asian recipients. We report on a Caucasian Australian patient with immunotherapy induced RCVS post heart transplantation to highlight the state of knowledge of the condition and the treatment dilemma it poses.

**Case presentation:**

A 51-year-old female underwent orthotopic heart transplantation at our institution. Induction immunotherapy comprised basiliximab, mycophenolate mofetil and methylprednisolone. On day 6 post-transplantation the patient was transitioned to oral prednisolone and tacrolimus. On day 7 the patient began to experience bilateral, severe, transient occipital and temporal headaches. On day 9 tacrolimus dose was up-titrated. A non-contrast computed tomography brain (CTB) was normal. Endomyocardial biopsy on day 12 demonstrated moderate Acute Cellular Rejection (ACR), which was treated with intravenous methylprednisolone. That evening the patient experienced a 15-minute episode of expressive dysphasia. The following morning she became confused, aphasic, and demonstrated right sided neglect and right hemianopia. A CT cerebral perfusion scan demonstrated hypoperfusion in the left middle cerebral artery (MCA) territory and cerebral angiography revealed widespread, focal multi-segmental narrowing of the anterior and posterior circulations. A diagnosis of RCVS was made, and nimodipine was commenced. As both steroids and tacrolimus are potential triggers of RCVS, cyclosporin replaced tacrolimus and methylprednisolone dose was reduced. A further CTB demonstrated a large left MCA territory infarct with left M2 MCA occlusion. The patient made steady neurological improvement. She was discharged 34 days post-transplantation with mild residual right lower limb weakness and persistent visual field defect on verapamil, cyclosporine, everolimus, mycophenolate mofetil and prednisolone.

**Conclusion:**

Reversible cerebral vasoconstriction syndrome is rare after orthotopic heart transplantation. Until now, RCVS has been almost exclusively described in Asian recipients, and is typically caused by immunotherapy. The condition may lead to permanent neurological deficits, and in the absence of definitive treatments, early recognition and imaging based diagnosis is essential to provide the opportunity to remove the causal agent(s). Co-existent ACR, can pose unique treatment difficulties.

## Introduction

Reversible cerebral vasoconstriction syndrome (RCVS) typically manifests as severe recurrent thunderclap headaches (TCH), may be accompanied by other neurological symptoms and signs, and is characterized radiologically by diffuse, multifocal segmental narrowing of cerebral arteries. Seizures, subarachnoid haemorrhage (SAH), posterior reversible encephalopathy syndrome (PRES), intracerebral haemorrhage (ICH) and ischaemic stroke (IS) are recognised complications. The clinical and radiological features of RCVS are dynamic which makes accurate diagnosis difficult and stroke may occur several days after normal imaging. Most patients with RCVS recover fully within 3 months, however up to 10% of patients are left with permanent deficits, with death a rare outcome [1–3]. While RCVS may be idiopathic, causal conditions or factors are identified in up to 60% of cases, and include exposure to vasoactive drugs and the use of immunosuppressants [1–6]. There have been a few reports of RCVS following heart transplantation, predominantly in Asian recipients, [7–11] resulting in residual neurologic deficits and requiring modification of both maintenance and acute rejection immunosuppressive therapy. We report on a Caucasian Australian patient with immunotherapy induced RCVS post heart transplantation to highlight the present state of knowledge of the condition and the treatment dilemma it poses in this cohort of patient. This case is reported according to CARE guidelines [12].

## Case report

A 51-year-old female underwent orthotopic heart transplantation for hypertrophic cardiomyopathy at our institution. The patient had a prior history of surgical septal myomectomy and AICD insertion and reported New York Heart Association (NYHA) Functional Classification IV symptoms. In addition, she had cardio-renal syndrome with a baseline serum creatinine of 119umol/L, eGFR 45 mL/min/1.73m^2^. The patient had an uncomplicated surgical procedural and intensive-care (ICU) stay with low-dose isoprenaline 0.5mcg/min and noradrenaline (maximal rate 12mcg/min) which were both weaned within 24 h of the operation. Induction immunotherapy comprised basiliximab, mycophenolate mofetil and methylprednisolone. Tacrolimus was not initially prescribed due to a rise in creatinine to 210umol/L. Instead, anti-thymocyte globulin (ATG) 125 mg/daily was administered over a 3-day period commencing day 3 post-transplantation. On day 6 the patient was transitioned to oral prednisolone and tacrolimus 1.5 mg twice daily after a return to baseline creatinine.

**Fig. 1:**
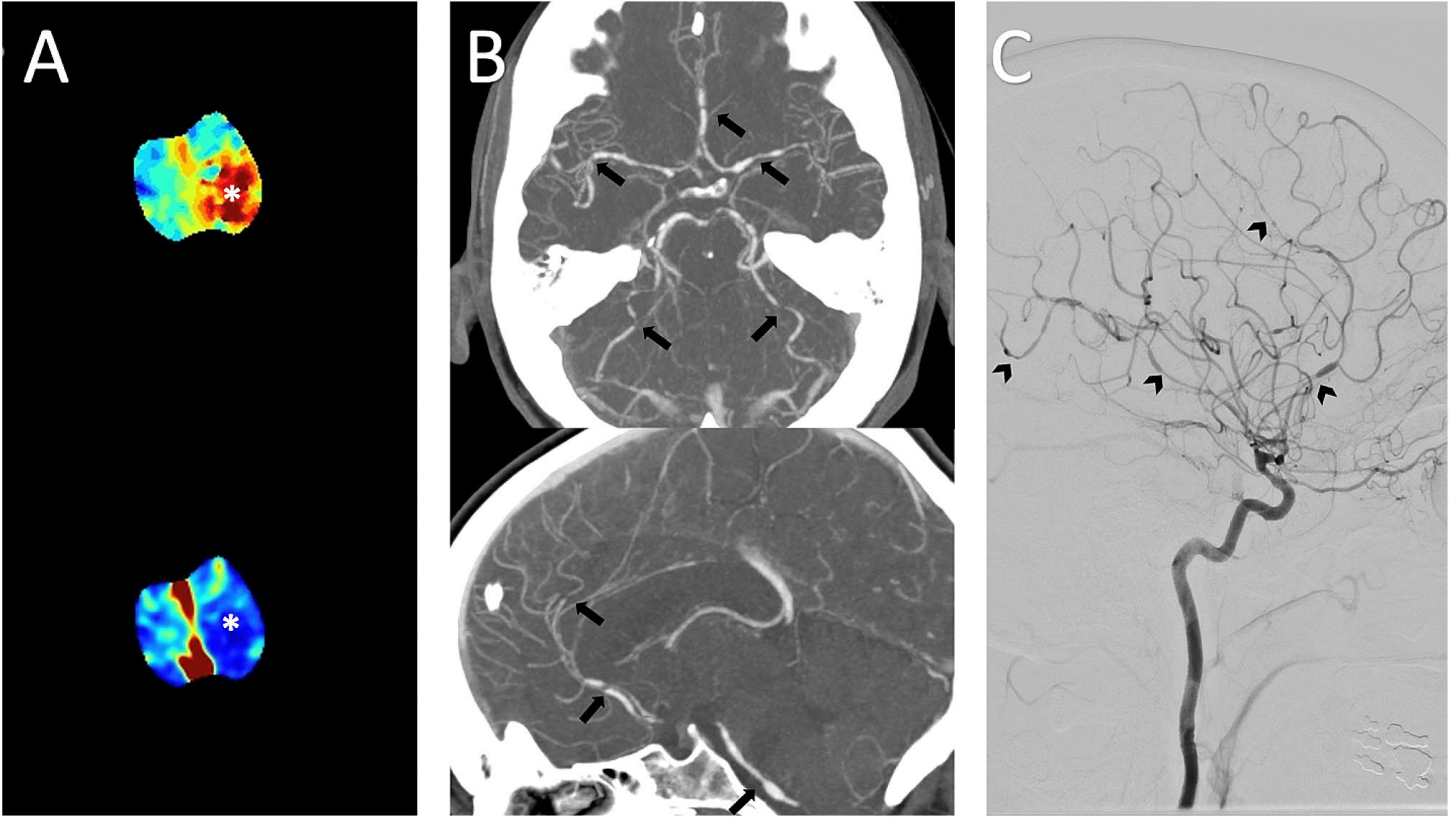
Initial imaging **A**: Axial CT perfusion Tmax (top) and cerebral blood flow (bottom) slices at the vertex demonstrated left-sided acute ischaemic penumbra and infarct core (*) **B**: CT angiogram maximum intensity projections (MIPs) axial (top) and sagittal (bottom) showed bilateral multifocal stenoses and beading (arrows) of the anterior and posterior circulations **C**: Interventional angiography of the left internal carotid artery sagittal projection confirmed similar findings (arrow heads)

On day 7 post-transplantation the patient began to experience bilateral, severe, transient occipital and temporal headaches. On day 9 tacrolimus dose was up-titrated to 3 mg twice daily then 4 mg twice daily the following day, aiming to achieve a therapeutic tacrolimus level. Due to recurrent headaches a non-contrast computed tomography brain (CTB) was performed and was normal. Her second endomyocardial biopsy on day 12 demonstrated International Society for Heart and Lung Transplantation (ISHLT) grade 2 R (moderate) Acute Cellular Rejection (ACR) for which the patient received methylprednisolone 1gm intravenously (IV) with plans for further 500 mg IV pulses the following two days. Tacrolimus trough level at this time was 5.7ug/L. That evening the patient experienced a 15 minute episode of expressive dysphasia. A repeat non-contrast CTB revealed only early grey-white matter differentiation in the left parietal region. The following morning the patient became confused, aphasic, and demonstrated right sided neglect and right hemianopia. The patient’s National Institutes of Heath Stroke Scale (NIHSS) score [13] was 6 and blood pressure was 106/61mmHg. A CTB was again performed and remained unchanged. A CT cerebral perfusion scan (CTP), however, demonstrated hypoperfusion in the left middle and posterior cerebral artery (MCA-PCA) territories. Cerebral angiography (CTA) revealed widespread, bilateral focal multi-segmental narrowing of the anterior and posterior circulations with no large vessel occlusion. Figure 1 Neurology was consulted, a diagnosis of RCVS was made, and nimodipine 60 mg 4 hourly and aspirin 100 mg daily were commenced. Magnetic resonance (MR) cerebral imaging was not possible due to a retained AICD coil. In recognition that both steroids and tacrolimus are potential triggers of RCVS, cyclosporine was introduced in place of tacrolimus and the dose of IV methylprednisolone administered to treat the ACR was reduced to 250 mg. Following this the patient was transitioned to prednisolone 20 mg daily.

Due to fluctuating neurology with a worsening NIHSS score of 23 a further CTB was performed on day 14 post-transplantation and demonstrated a large evolving acute left MCA territory infarct with left M2 MCA occlusion. Levetiracetam was commenced empirically for seizure prophylxis. The patient made steady neurological improvement, with improvement of NIHSS score to 5. However, a repeat CTB/A performed on day 23 post-transplantation showed left parieto-occipital and right parieto-temporal hypoattenuation with diffuse small calibre circle of Willis vessels and only marginal improvement in the appearance of the anterior and middle cerebral arteries. The patient was commenced on additional immunotherapy with everolimus on day 27 with endomyocardial biopsy demonstrating ISHLT 0R. Furthermore, nimodipine was transitioned to verapamil prior to discharge to allow for an easier dosing schedule and frequent therapeutic drug monitoring was performed for cyclosporine and everolimus. She was discharged 34 days post-transplantation with mild residual right lower limb weakness and persistent visual field defect (NIHSS score 3) on verapamil 160 mg three times a day and immunosuppressant therapy comprising cyclosporine 150 mg twice daily (target level of 100–150 ug/L), everolimus 0.5 mg twice daily, mycophenolate mofetil 1.5 g twice daily and prednisolone 15 mg daily. Subsequent cardiac biopsies continued to demonstrate ISHLT 0R.

## Discussion

The true incidence of RCVS is unknown, is likely under diagnosed [1] and typically affects women in their fifth decade [2]. The clinical manifestations of RCVS usually follow an acute and self-limiting course with no new symptoms after 4 weeks [2]. More than 80% of patients present with recurrent TCH. Generalised tonic clonic seizures occur in up to 20% of patients [5] and focal neurological deficits from intracerebral haemorrhage or stroke are reported in 9 – 63% of cases [2, 14, 15]. The complications of RCVS tend to follow a defined time pattern. As exemplified in this case, ischaemic strokes are often bilateral, located in arterial watershed territories, and often occur at the end of the second week [2]. Computerised tomography and MR imaging may be initially negative in 30 – 70% of cases [1]. The anatomical basis and exact cause of RCVS remains unknown. Variability in the dense innervation of cerebral blood vessels with sensory afferents from the first division of the trigeminal nerve and dorsal root of C2 may explain the heterogeneity of vessels involved [1]. What is clear is that a sudden alteration of sympathetic control of cerebral vascular tone is central with endothelin-1, catecholamines and nitric oxide potentially contributing to the pathophysiology of vasoconstriction [1, 16]. Most RCVS cases post-transplantation, as illustrated by this case, have occurred within 2 weeks of transplantation (Table 1). In contrast, however, to the best of our knowledge, all previous RCVS cases confirmed by imaging have inexplicably occurred in Asian recipients. A likely case of RCVS in a Caucasian recipient was unable to be confirmed due to contrast allergy and the presence of epicardial pacing wires, precluding CTA and MR cerebral imaging respectively, prior to her death from sepsis [17]. Reported cases of RCVS do not seem to relate to the use of adrenergic agonists in the immediate post-operative period. Indeed, the leading causal factor is considered to be immunosuppressant pharmacotherapy, specifically, the use of tacrolimus [7–11], and not necessarily when at supratherapeutic levels [7, 18], rather than ATG or methylprednisolone. However, both of these agents have been associated with RCVS [19, 20]. Steroids may act by potentiating the vasoconstrictor effects of angiotensin II (AT II), norepinephrine and endothelin through upregulation of alpha-1 and AT II receptors [21]. The temporal relationship between the onset of RCVS, notably the focal neurologic sequelae, in this case potentially implicated both tacrolimus and methylprednisolone. It was for this reason that tacrolimus was immediately stopped and replaced with cyclosporine and that the dose of methylprednisolone was reduced. Steroid therapy was not discontinued given that the patient had biopsy proven moderate allograft rejection, however the dose given for ACR was substantially attenuated. Additionally, everolimus was added as an adjunct to immunosuppressive therapy, to allow for a more rapid wean of oral prednisolone.

**Table 1:** Cases of Tacrolimus-induced RCVS confirmed by imaging following heart transplantation

Authors	Age	Gender	Race	Presenting symptoms	Onset (POD)	Elevated tacrolimus level	Management	Allograft rejection	Permanent neurological deficit	Clinical improvement (POD)	Resolution of vasoconstriction (POD)	Cerebral infarction
Kodama et al [7]	15	F	Asian	Seizure	8	Yes	Tacrolimus ceased	No	No	Complete, 11	Complete, 11	No
Ban et al [8]	52	M	Asian	Seizure	8	NA	Tacrolimus dose reduced, later ceased	No	Yes	Not specified	Complete, 90	Yes: bilateral fronto-parietal-occipital
Kumai et al [9]	47	F	Asian	Headache	11	NA	Tacrolimus ceased Basiliximab/Everolimus commenced	Yes	No	Complete, 27	Complete, 27	No
Park et al [10]	50	M	Asian	Seizure	4	No	Tacrolimus dose reduced, later ceased	No	Yes	Not specified	Residual focal arterial spasm, 60	Yes: fronto-parietal
Maeda et al [18]	48	F	Asian	Headache	11	Yes	Tacrolimus ceased Basiliximab/Everolimus commenced	No	No	Complete, 12	Complete, 12	Yes: bilateral temporal and occipital lobes
Inoue et al [26]	44	F	Asian	Headache	6	No	Tacrolimus ceased Cyclosporin commenced	No	No	Complete, 6	NA	No
Tsukahara et al [27]	13	M	Asian	Headache	7	No	Tacrolimus ceased Cyclosporin commenced	No	No	Complete, 14	Improved, 14 Complete, 90	No

*POD: Post-operative day, NA: Not available

In the absence of randomised controlled trials of treatment in RCVS to guide therapy, and having identified and removed the precipitating factors, we administered drugs targeting vasospasm, specifically the calcium channel blockers nimodipine and verapamil. There is, however, no proven efficacy of nimodipine in reducing the time course of cerebral vasoconstriction or prevention of haemorrhagic or ischaemic complications in RCVS [2, 15, 22]. The use of verapamil in this setting is complicated by its inhibition of CYP3A4 enzyme which can lead to increased plasma concentration of both cyclosporine and everolimus. The dose of cyclosporine and everolimus was down-titrated and therapeutic drug monitoring was performed to ensure levels remained within therapeutic target. In addition, while direct intra-arterial administration of nimodipine [23], verapamil [24] or milrinone [25], and intracranial angioplasty [23] have been reported to have had some success in this setting, we chose not to employ these therapies. This was due to the inherent risks, including arterial dissection or perforation, and reperfusion injury, and because of the diffuse nature of the pathological process.

Reassuringly, the patient in question has been left with mild permanent neurologic deficits including mild right sided visual inattention and neglect, occasional word finding difficulty, reduced right hand dexterity and very mild right leg pyramidal weakness (NIHSS 2, modified Rankin score 2). Further she is unlikely to have a recurrence [1, 2] and has a normally functioning cardiac allograft. The patient remains on verapamil 160 mg TDS. Prednisolone has been weaned to 5 mg daily with an intention to cease.

**Fig. 2:**
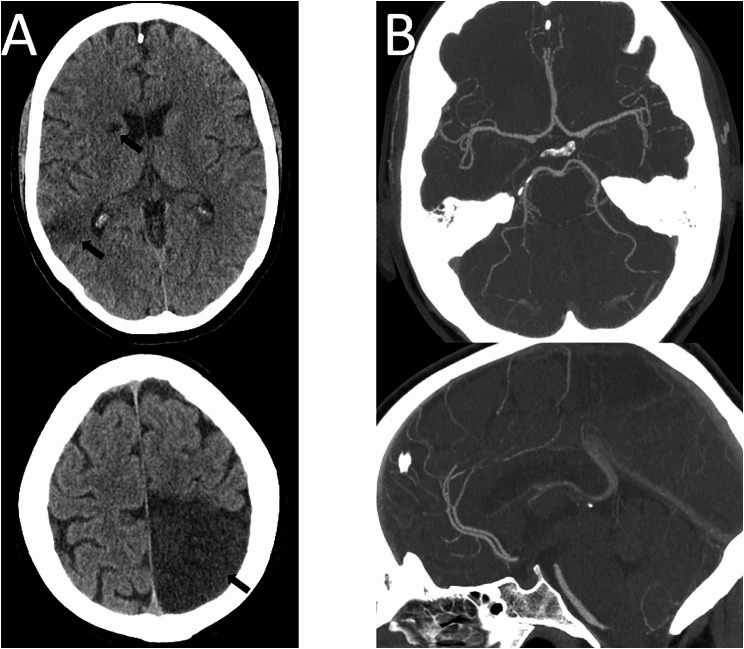
Follow up imaging **A**: Non contrast CT axial slices showing established infarcts in the right caudate, right temporoparietal and left parietal regions (arrows) **B**: CT angiogram MIPs axial (top) and sagittal (bottom) showing complete resolution of previous multifocal stenoses

At 6 months post-transplantation, CTB has demonstrated established areas of infarction in the left parieto-occipital, right caudate and parietotemporal areas with no new or subacute infarction. CTA has shown an improved appearance of the circle of Willis vessels with normalization of the basilar and posterior circulation vessels and only residual minor diffuse irregularity of the more distal vessels with no high-grade stenoses Figure 2.

## Conclusion

Reversible cerebral vasoconstriction syndrome is rare after orthotopic heart transplantation and is typically caused by immunotherapy. Noteworthy, until now, RCVS has almost exclusively and inexplicably been documented in Asian heart transplant centres and should be subject to further research. Although frequently benign, RCVS may lead to permanent neurological deficits, and in the absence of definitive treatments, early recognition and imaging based diagnosis is essential in order to provide the opportunity to remove the causal agent(s). In the presence of co-existent ACR, this can pose unique treatment difficulties for the transplant team.

## Data Availability

No datasets were generated or analysed during the current study.

## Abbreviations

RCVS: Reversible Cerebral Vasoconstriction Syndrome
PRES: Posterior Reversible Encephalopathy Syndrome
TCH: Thunderclap Headaches
SAH: Subarachnoid Haemorrhage
ICH: Intracerebral Haemorrhage
IS: Ischaemic Stroke
NIHSS: National Institutes of Health Stroke Scale
MCA: Middle Cerebral Artery
CTB: Computed Tomography Brain
CTP: Cerebral Perfusion Scan
CTA: Cerebral Angiography
MR: Magnetic Resonance
ISHLT: International Society for Heart and Lung Transplantation
ACR: Acute Cellular Rejection
ATG: Anti-Thymocyte Globulin
NYHA: New York Heart Association
AICD: Automatic Implantable Cardiac Defibrillator
